# Case Report: Angioimmunoblastic T-cell lymphoma initially diagnosed as eosinophilic granulomatosis with polyangiitis

**DOI:** 10.3389/fmed.2025.1716129

**Published:** 2026-01-22

**Authors:** Yingmeng Ni, Yidan Sun, Simin Xie, Jialing Xie, Guochao Shi, Ranran Dai, Yi Guo

**Affiliations:** 1 Department of Pulmonary and Critical Care Medicine, Ruijin Hospital, Shanghai Jiao Tong University School of Medicine, Shanghai, China; 2 Institute of Respiratory Diseases, Shanghai Jiao Tong University School of Medicine, Shanghai, China; 3 Department of Pathology, Ruijin Hospital, Shanghai Jiao Tong University School of Medicine, Shanghai, China

**Keywords:** AITL, asthma, EGPA, lymphoma, misdiagnose

## Abstract

**Background:**

Eosinophilic granulomatosis with polyangiitis (EGPA) and angioimmunoblastic T-cell lymphoma (AITL) are distinct entities that can present with overlapping clinical features, posing a significant diagnostic challenge. This case highlights a critical diagnostic pitfall where AITL was initially misdiagnosed as EGPA.

**Case presentation:**

A 55-year-old man presented in 2021 with recurrent wheezing, dyspnea, chronic sinusitis, peripheral eosinophilia (7.35 × 10⁹/L), and skin rash, leading to an initial diagnosis of asthma and later EGPA. Despite treatment with systemic corticosteroids, omalizumab, and mepolizumab, his respiratory symptoms persisted, and lymphadenopathy progressed. A fine-needle lymph node biopsy initially suggested Kimura disease. In 2024, the appearance of a parotid mass prompted further investigation. A subsequent surgical lymph node biopsy in 2025 revealed an effaced nodal architecture with a proliferation of atypical T-cell positive for CD3, CD5, ICOS, and PD-1. T-cell receptor gene rearrangement demonstrated clonality, and Epstein–Barr virus-encoded RNA was detected in situ. These findings confirmed the diagnosis of AITL. Treatment was switched to the CHOP chemotherapy regimen, leading to symptomatic improvement and normalization of eosinophil counts.

**Conclusion:**

This case underscores that AITL can closely mimic EGPA, presenting with severe asthma, hypereosinophilia, sinusitis, and systemic symptoms. It emphasizes the necessity of considering underlying lymphoma in patients with suspected EGPA who show an atypical or refractory course to conventional therapy. A definitive diagnosis often requires an adequate tissue sample, preferably from a surgical lymph node biopsy, to avoid misdiagnosis and ensure appropriate management. Respiratory physicians should maintain a high index of suspicion for lymphoid malignancies in such complex presentations.

## Introduction

Eosinophilic granulomatosis with polyangiitis (EGPA) is a rare systemic vasculitis characterized by asthma, chronic rhinosinusitis, peripheral blood eosinophilia, and necrotizing vasculitis. The diagnostic journey is often complex, as its initial presentation, particularly treatment-resistant asthma with hypereosinophilia, can overlap with other conditions such as hypereosinophilic syndrome (HES) and, more rarely, lymphoid malignancies. Angioimmunoblastic T-cell lymphoma (AITL), a subtype of peripheral T-cell lymphoma, is notorious for its paraneoplastic manifestations, which frequently mimic autoimmune or inflammatory disorders, including rash, arthralgia, and hypergammaglobulinemia. The co-occurrence of eosinophilia, while not a classic feature, further blurs the diagnostic boundaries. Herein, we report a challenging case of a patient whose clinical picture was highly suggestive of EGPA but was ultimately diagnosed with AITL, illustrating a critical diagnostic pitfall and emphasizing the necessity of considering underlying lymphoma in refractory cases with eosinophilic predominance.

## Case report

### Initial presentation (2021)

A 55-year-old man has experienced recurrent intermittent episodes of chest tightness, shortness of breath, and wheezing, accompanied by coughing with expectoration and severe nasal congestion since 2021. He initially presented to a local hospital and underwent chest computed tomography (CT), which revealed multiple areas of bronchial wall thickening with the formation of mucus plugs. Spirometry demonstrated obstructive ventilatory impairment and markedly elevated fractional exhaled nitric oxide (FeNO) 215 ppb. Blood tests revealed eosinophilia (3.41*10^9^ cells/L) and an increased IgE level (116 IU/mL). Based on the above examinations, a preliminary diagnosis of asthma was made. He received budesonide and formoterol fumarate powder for inhalation, omalizumab, and leukotriene receptor antagonists as initial therapies. However, his symptoms did not improve.

### First referral (2023) and initial diagnoses

The patient was transferred to our hospital for further evaluation in 2023. On admission, in addition to the above symptoms, he also experienced hearing loss and developed rashes after eating seafood. Blood tests revealed eosinophilia (7.35*10^9^cells/L). A PET/CT scan was performed, showing tonsil enlargement with increased metabolism and multiple lymph nodes with mildly elevated metabolism, spleen enlargement, and pan-sinusitis. The pathological findings after sinusitis surgery indicated a few scattered eosinophils in the nasal mucosal tissue. Bone marrow aspiration revealed eosinophils accounting for 5–10% of the total nucleated cells, and the pathology of the lymph node needle puncture biopsy showed a lymphoid proliferative lesion with mild atypia and numerous eosinophils, consistent with a diagnosis of “Kimura disease.” Regular follow-up examinations are recommended according to consultation with a hematologist. Taken together, the patient was therefore rediagnosed with Kimura disease and eosinophilic granulomatosis with polyangiitis (EGPA), according to the 1990 ACR diagnostic criteria. Budesonide/glycopyrronium bromide/formoterol for inhalation, systemic corticosteroids (1 mg/kg/day), omalizumab (600 mg q2w), and mepolizumab (100 mg q4w) were administered.

### Treatment failure and new symptoms (2024)

**Figure 1:**
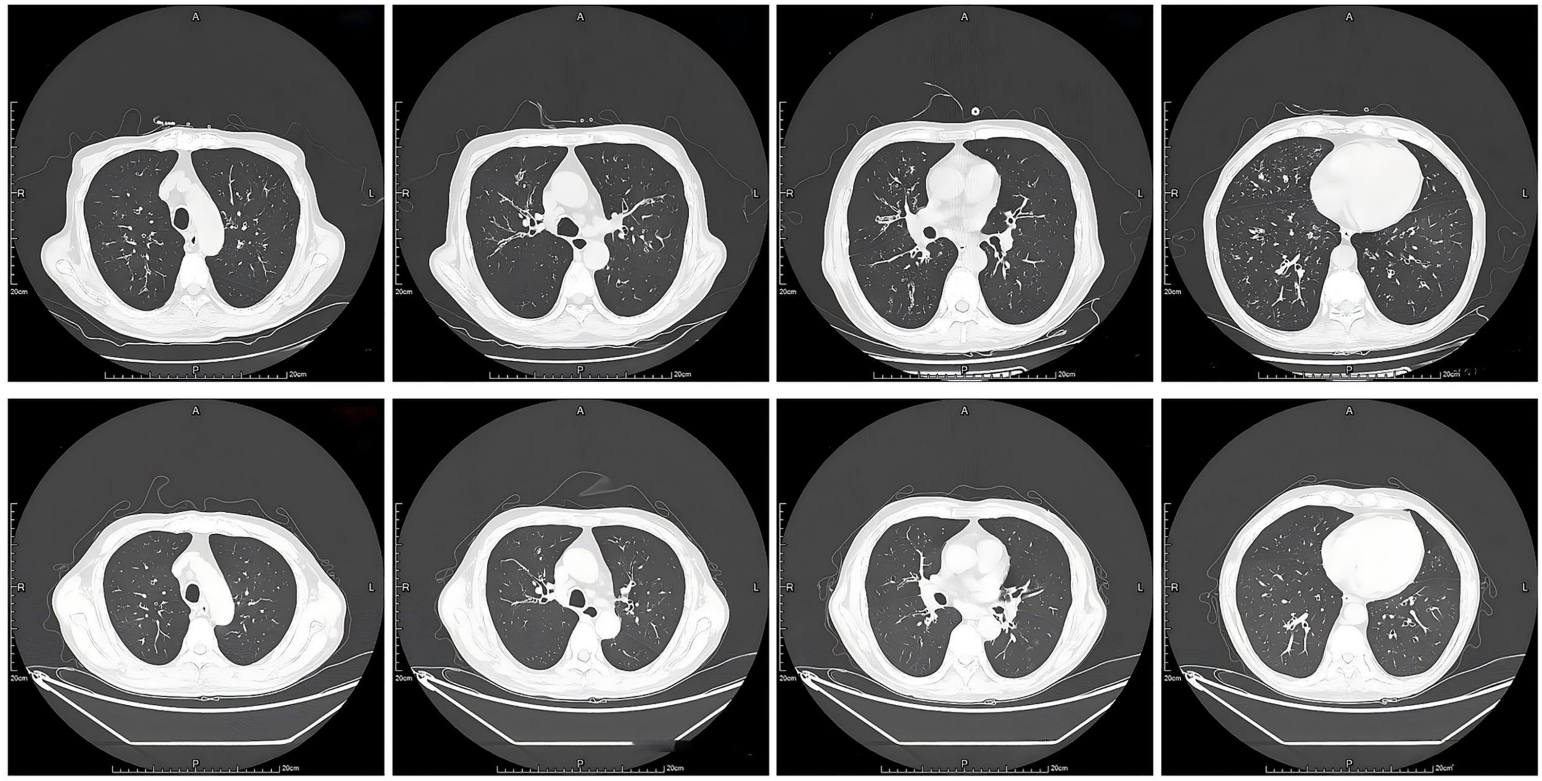
Chest CT scan. Top, chest CT scan in September 2024; bottom, chest CT scan in February 2025.

After treatment, his eosinophil count decreased, but his respiratory manifestations were not ameliorated, and the lymphadenopathy did not subside. The patient was readmitted to our hospital due to the appearance of a parotid gland mass in August 2024. Biopsy of the mass was performed, and the histopathological finding was basically consistent with that of the lymph node biopsy. A second lymph node puncture biopsy was performed, and the pathology showed T cell hyperplasia predominantly with mild morphological atypia and TCR clonal bands found, but a low Ki67 expression level (40%). After consultation with a rheumatologist, intravenous cyclophosphamide (0.8 g q4w) was administered. The patient was under regular mepolizumab and CTX treatment from July 2024 to November 2024, and the peripheral eosinophil count was kept normal, but the symptoms fluctuated, and a progressive thickening of the airway wall was noticed on the chest CT scan (Figure 1). Thus, dupilumab was administered in December 2024, and mepolizumab was stopped. Dupilumab (300 mg q2w) was given alternately with omalizumab (600 mg q2w) for 3 months. During this period, the patient’s wheezing symptoms were slightly improved, lung function was improved, and bronchial dilation and airway wall thickening on the CT scan improved as well.

### Definitive diagnosis (2025)

**Figure 2:**
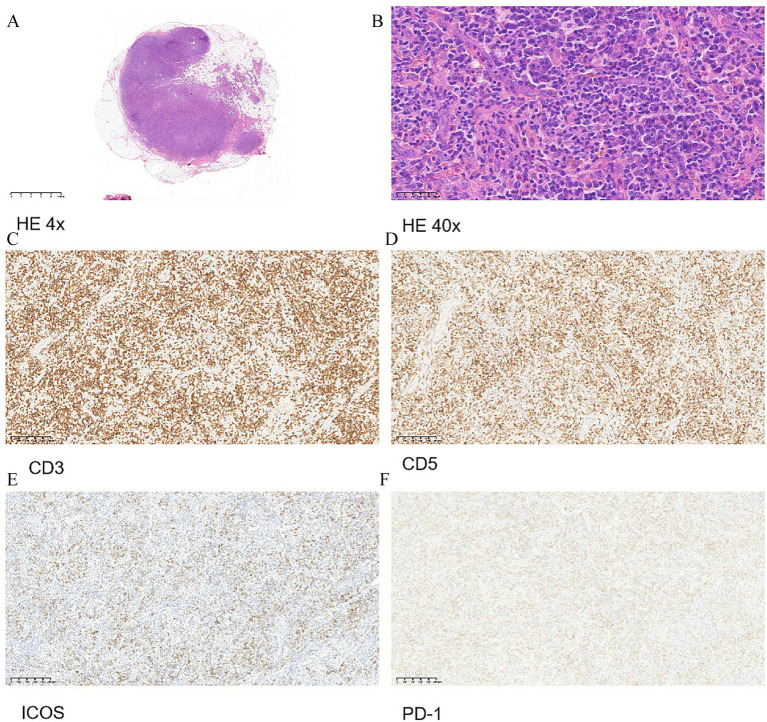
Pathology and immunohistochemistry of lymph node surgical biopsy. **(A)** HE 4x. **(B)** HE 40x. **(C)** Immunohistochemistry of CD3. **(D)** Immunohistochemistry of CD5. **(E)** Immunohistochemistry of ICOS. **(F)** Immunohistochemistry of PD-1.

In February 2025, the patient developed a rapid enlargement of superficial lymph nodes, leading to difficulty turning the neck, accompanied by the re-elevation of peripheral blood eosinophils (7.35*10^9^ cells/L). Thus, dupilumab was stopped. Since the patient’s wheezing improved, he tolerated a surgical lymph node biopsy, which was a decisive examination. The pathology of the surgical biopsy completed showed lymphoproliferative lesions with a basic structure of lymph nodes blurred; immunohistochemical markers showed that the hyperplastic cells were mainly T lymphocytes (CD3+, CD5+, CD7+, ICOS+, PD-1+), with mild morphological atypia; the TCR gene rearrangement test showed clonal bands; Epstein–Barr virus-encoded RNA *in situ* hybridization was positive in a certain proportion of tumor cells (Figure 2). These pathological performances were consistent with peripheral T-cell lymphoma, predisposed to lymph nodes, follicular helper T-cell lymphoma, vascular immunoblast type. The patient was finally diagnosed with angioimmunoblastic T cell lymphoma (AITL) and was treated with the CHOP regimen on 5 March 2025. After several cycles of chemotherapy, the EOS level of the patients still decreased with the interruption of mepolizumab therapy, and the wheezing symptoms were further improved. The patient is now being followed up in the hematology department.

## Discussion

The patient was initially misdiagnosed with asthma, which is a common airway inflammatory disease, but asthmatic symptoms can also be the result of other systemic diseases. EGPA is one of the differential diagnoses for asthma (1). EGPA, formerly known as Churg–Strauss syndrome, is a rare but serious form of vasculitis characterized by the inflammation of small- to medium-sized blood vessels and significant eosinophilia. In our case, the patient showed typical asthmatic symptoms, chest ground-glass opacities, pan-sinusitis, rashes with hypereosinophilia, and was rediagnosed with EGPA after the failure of asthma treatment. However, he did not respond well to corticosteroids and mepolizumab. This led us to dig out the underlying cause of his “asthmatic symptoms.”

**Figure 3:**
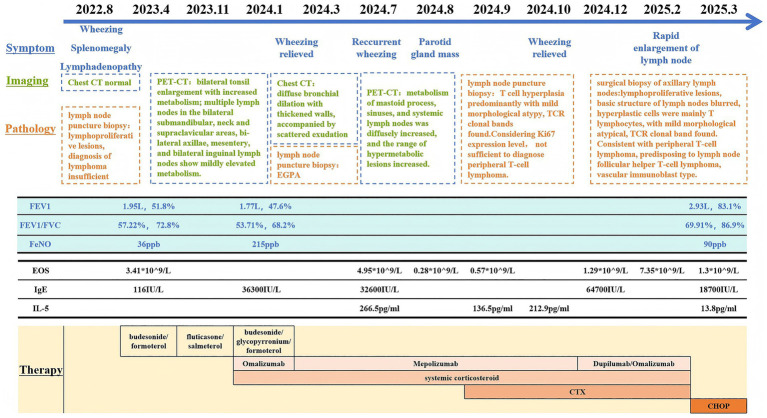
Timeline of clinical presentation and work-up. PET, Positron emission tomography; FEV1, Forced expiratory volume in 1 s; FVC, Forced vital capacity; FeNO, Fractional exhaled nitric oxide; EOS, Eosinophils; CTX, Cyclophosphamide.

There is considerable overlap in clinical presentations between hypereosinophilia syndrome (HES) and EGPA. Distinguishing between the two can be challenging. HES is a rare and complex hematological disorder characterized by persistently elevated levels of eosinophils (>1,500 cells/mL) (2). The pathophysiology of HES is rooted in both primary and secondary causes of eosinophilia. Primary causes of HES are often associated with hematologic malignancies or myeloproliferative disorders (Figure 3 and Table 1).

**Table 1:** Treatment responses.

Treatment	Change of clinical manifestation	Change of test results
Budesonide/Formoterol/Fumarate Powder + systematic corticosteroid	Wheezing worsened, lymphadenopathy worsened	Eosinophil count elevated, IgE elevated, Spirometry worsened, Chest CT: almost normal
Omalizumab	Wheezing worsened, lymphadenopathy worsened, new symptom: rash	Eosinophil count elevated, IgE elevated, Spirometry worsened, Chest CT:diffuse bronchial dilation with thickened walls, accompanied by scattered exudation
Mepolizumab	Wheezing recurrent, lymphadenopathy stable, new symptom: parotid gland mass	Eosinophil count decreased, IgE elevated, Spirometry did not check, Chest CT: progressive thickening of airway wall
Mepolizumab + CTX	Wheezing ameliorated, lymphadenopathy stable, rash subsided, parotid gland mass subsided	Eosinophil count decreased to normal, IgE elevated, Spirometry did not check, Chest CT: progressive thickening of airway wall
Dupilumab	Wheezing ameliorated, but rapid enlargement of lymph node	Eosinophil count re-elevated, IgE kept high, Spirometry improved, Chest CT: bronchial dilation and airway wall thickening improved
Chemotherapy	Wheezing ameliorated, lymphadenopathy ameliorated	Eosinophil count decreased to normal, IgE decreased, Spirometry improved, Chest CT: almost normal

The first-line treatment for HES typically involves systemic corticosteroids. Hydroxyurea and imatinib may also be administered, particularly in steroid-refractory cases (3). Research into new treatment modalities is ongoing. Mepolizumab has recently received FDA approval for treating patients aged 12 years and older with HES (4). Benralizumab and dupilumab were also reported to be used in HES patients (5, 6). In this case, our patient’s eosinophil count decreased with mepolizumab, but the symptoms and radiological presentation did not ameliorate. The decrease of eosinophils allowed us to try dupilumab, which should not be used when the eosinophil count is high, and the patient showed a greater response to dupilumab with amelioration of clinical symptoms, spirometry, and chest CT imaging as well. However, the patient showed a quick enlargement of the lymph node and a dangerous re-elevation of peripheral eosinophil count after several injections of dupilumab. It is not clear whether this was related to dupilumab administration.

The patient was finally diagnosed with angioimmunoblastic T cell lymphoma (AITL) by a surgical lymph node biopsy. Clinical symptoms of AITL frequently mimic those of autoimmune or inflammatory disorders, leading to diagnostic challenges and delayed treatment. In our case, based on the initial fine-needle biopsy results, the diagnosis was considered to be Kimura’s disease. However, in retrospect of the entire disease course, the extensive eosinophil infiltration observed in the initial fine-needle aspiration should actually be attributed to the massive proliferation of Tfh cells in AITL and the release of cytokines such as IL-5 and IL-13. The small tissue sample from the fine-needle aspiration biopsy could lead to misinterpretation. There is also the possibility that the progression of the disease itself caused the change in pathological results between the two biopsies. Thus, surgical lymph node biopsy should be performed whenever possible.

One significant aspect of AITL is the involvement of the Epstein–Barr virus (EBV) (7), which is detectable in most patients, and also in our case. The precise role of EBV in AITL development remains unclear; however, it is believed to influence T cell activation and promote the survival of infected cells, thereby aiding in the progression of the disease. Profound immunosuppression due to the progression of AITL induces EBV reactivation in B cells. It is reported that EBV-positive B cells are frequently detected by AITL. Regrettably, we were not able to confirm whether EBV + cells were T cells or B cells in our case.

There are a few reports about misdiagnosis between AITL and EGPA (8, 9). Le Roy et al. (8) reported a 55-year-old woman, who complained of approximately a 1-year history of dry cough and dyspnea evolving into acute exacerbation. Initial blood revealed eosinophilia, and a chest CT-scan showed bilateral reticulonodular and ground-glass opacities. She was diagnosed with EGPA. Her symptoms were partially improved with combined therapies but relapsed after 2 months of follow-up. PET/CT scan showed homogeneous bone marrow and thyroid 18F-FDG uptake, cervical, diffuse hypermetabolic lymph nodes. The biopsy finally revealed AITL. The authors suggested that AITL may mimic EGPA and highlighted the multifaceted clinical presentation and the wide spectrum of other autoimmune manifestations that AITL may mimic. Underlying T-cell lymphoma should be considered in patients with suspected EGPA. Interestingly, Kawauchi et al. (9) reported EGPA may conversely mimic peripheral T-cell lymphoma. His patient was initially diagnosed with lymphoma via partial intestinal resection due to small intestinal perforation but recurred with an elevated eosinophil count. Additional review of the resected ileum revealed fibrinoid necrosis, eosinophilic infiltration, and rupture of the internal elastic laminae in the small- and medium-sized arteries of the submucosal layer, indicating necrotizing vasculitis, and a clonal T-cell population with TCR *β* gene rearrangement was not detected. Finally, the patient was therefore rediagnosed with EGPA.

Lymphoma is known to be associated with autoimmune diseases, probably related to persistent antigenic stimulation and immunosuppressive medications. Some people believe that B- and T-cell hyperactivity on account of chronic antigenic stimulation seems to play a key role in the pathogenesis of lymphoma. Advanced age at diagnosis, prolonged disease course, and disease severity are thought to increase the risk of lymphoma development in patients with autoimmune disorders, which may be induced by EGPA (10).

In conclusion, we experienced a case of AITL perfectly mimicking EGPA with recurrent asthmatic symptoms, chest ground-glass opacities, pan-sinusitis, rashes, and hypereosinophilia. We report this case to remind respiratory physicians that a thorough work-up and close follow-up are essential for patients with EGPA to eliminate underlying lymphoma, especially when they do not respond well to classic treatments.

## Data Availability

The original contributions presented in the study are included in the article/supplementary material, further inquiries can be directed to the corresponding authors.
